# Role of the Transcription Factor CREB in Ethanol-Induced Endoplasmic Reticulum Stress and Apoptosis in PC12 Cells

**DOI:** 10.3390/biology14091277

**Published:** 2025-09-16

**Authors:** Marica Németh, Barbara Brandt, Hajnalka Les, Petra Kele-Morvai, András Maifeld, Tibor A. Rauch, Kristóf Schwartz, Marianna Pap

**Affiliations:** 1 Department of Medical Biology, Medical School, University of Pécs, 7624 Pécs, Hungary; marica.nemeth@aok.pte.hu (M.N.); bb.barbara91@gmail.com (B.B.); hajnalka.horvath@aok.pte.hu (H.L.); petra.morvai@aok.pte.hu (P.K.-M.); 2 Charité Research Organisation, 10117 Berlin, Germany; andras.maifeld@charite-research.org; 3 Department of Biochemistry and Medical Chemistry, Medical School, University of Pécs, 7624 Pécs, Hungary; tibor.rauch@aok.pte.hu; 4 Department of Psychology and Health Management, Faculty of Health and Sport Sciences, Széchenyi István University, 9026 Győr, Hungary; schwartz.kristof@ga.sze.hu

**Keywords:** ER stress, apoptosis, CREB, ethanol, PC12 cells

## Abstract

Ethanol is a known neurotoxic agent that induces endoplasmic reticulum (ER) stress and apoptosis in nerve cells. The transcription factor CREB is crucial for cell survival under stress; however, its involvement in ethanol-induced endoplasmic reticulum (ER) stress remains poorly understood. We examined the effects of ethanol on wild-type PC12 cells and CREB-overexpressing PC12-CREB cells. Cell viability was evaluated by ATP assays, apoptosis was detected by Hoechst staining, and key proteins involved in ER stress and apoptotic signaling were analyzed by Western blot analysis. Ethanol treatment decreased cell viability and increased apoptosis in wild-type PC12 cells in a time-dependent manner. In contrast, PC12-CREB cells-maintained viability and showed significantly lower apoptotic cell numbers. Ethanol activated markers of ER stress (BiP, CHOP, ATF6) and pro-apoptotic pathways (phosphorylation of JNK and p38 MAPK) in wild-type cells. In CREB-overexpressing cells, CHOP induction and JNK activation were decreased, while the expression of the anti-apoptotic protein Mcl-1 was increased. CREB overexpression protects against ethanol-induced ER stress and apoptosis. This protective effect is mediated through modulation of unfolded protein response (UPR) signaling and regulation of pro-and anti-apoptotic gene expression. These findings underscore a potential role for CREB in attenuating ethanol-induced neurotoxicity.

## Simple Summary

Alcohol has the potential to induce cellular damage in various organs, including the brain. This damage occurs through cellular stress, which disrupts protein synthesis within the endoplasmic reticulum. Such stress can ultimately lead to cell death. In this study, we employed PC12 cells, a neuronal cell line, to investigate the mechanisms underlying this process and assess whether the natural protein CREB offers protective effects against alcohol-induced toxicity. We found that normal cells were killed by alcohol treatment, but cells with higher levels of CREB protein survived much better. The CREB protein mitigated the cellular stress response and activated protective genes, thereby reducing the likelihood of cell death. This means that CREB may help protect brain cells from the harmful effects of alcohol. Our findings may contribute to a deeper understanding of the mechanisms through which alcohol induces brain damage and provides insights into potential strategies for protection in the future.

## 1. Introduction

Alcohol is a widely used psychoactive and toxic substance. Data from the World Health Organisation (WHO) show that around 2.6 million deaths were caused by alcohol consumption in 2019 [1]. Alcohol has a crucial role in several diseases; it can influence the function of the immune system and the microbiome, and increase the risk of inflammation, cirrhosis, and tumor formation. Acute alcohol consumption influences the function of the central nervous system, disrupts cell homeostasis, and affects several signal transduction pathways [2].

During alcohol metabolism, highly reactive acetaldehyde is produced, which can induce DNA point mutations, double-strand breaks, and other structural changes in chromosomes. Furthermore, alcohol consumption induces the formation of reactive oxygen species (ROS), leading to oxidative stress, which contributes to ethanol-induced multi-organ damage [2]. The endoplasmic reticulum (ER) is responsible for the synthesis, folding, modification, and quality control of proteins. The alcohol-induced oxidative stress can lead to the formation of abnormally folded proteins. Unfolded and misfolded proteins accumulated in the ER lumen trigger the unfolded protein response (UPR) to maintain the equilibrium of the ER [3,4]. This mechanism decreases protein synthesis, increases the activity of the chaperone proteins to correct misfolded proteins, and attenuates the ER stress [4]. During UPR, three pathways are activated by the dissociation of the chaperone protein, glucose-regulated protein 78 (GRP78/BiP), from three sensor proteins: protein kinase RNA-like ER kinase (PERK), inositol-requiring enzyme 1 (IRE1), and activating transcription factor 6 (ATF6).

During UPR, BiP dissociates from PERK, leading to autophosphorylation and dimerization of the transmembrane protein. The activated PERK phosphorylates the eukaryotic translation initiation factor 2 (eIF2α), repressing the initiation of translation. Under ER stress, the eIF2α enhances the expression of activating transcription factor 4 (ATF4), inducing C/EBP-homologous protein (CHOP) transcription [5]. The transcription factor CHOP induces apoptosis by diminishing the expression of anti-apoptotic and enhancing the levels of pro-apoptotic Bcl-2 protein family members [6].

Dissociation of the BiP protein leads to autophosphorylation of the IRE1 protein and activation of its kinase and ribonuclease activities. The activated IRE1 cleaves the transcription factor X-box binding protein (XBP1) mRNA; thus, the activated spliced XBP1 regulates the expression of chaperone proteins and ER-associated degradation (ERAD) associated proteins (e.g., CHOP) [6]. The activated IRE1 phosphorylates the stress kinase, Jun N-terminal kinase (JNK), leading to the expression of Bim pro-apoptotic and Bcl-2 anti-apoptotic proteins [3]. The p38 mitogen-activated protein kinase (p38 MAPK) is also activated by IRE1, stimulating the phosphorylation of pro-apoptotic proteins [6].

The transmembrane protein, ATF6 possesses a leucine-zipper transcription factor domain, which is cleaved under ER stress and regulates the expression of UPR genes (XBP1, CHOP, GRP78/BiP) [5].

Members of the Bcl-2 protein family play a crucial role in the ER stress-induced apoptosis. The multi-domain pro-apoptotic proteins (e.g., Bak, Bax, Bok) form pores in the outer membrane of the mitochondrion to increase its permeability and mediate cytochrome c release. The Bcl-2 protein family members interact directly or indirectly with each other and influence their activity. Anti-apoptotic proteins (e.g., Bcl-2, Mcl-1, Bcl-xL, Bcl-w) bind to multi-domain proteins and limit their activity [7]. Pro-apoptotic BH3-only proteins (e.g., Bim, Bid, Puma) exhibit high-affinity binding to all anti-apoptotic proteins, positioning them as potent initiators of apoptosis. Anti-apoptotic Bcl-2 proteins can be neutralized by BH3-only proteins; thus, multi-domain pro-apoptotic proteins can form channels in the mitochondrial membrane [8]. Cytochrome c release leads to the formation of the apoptosome and the activation of caspase-9 proteins, which are the executors of apoptosis [9].

Cyclic AMP (cAMP)-response element-binding protein 1 (CREB) belongs to the family of leucine zipper transcription factors; its basic leucine zipper (bZIP) DNA binding domain recognizes the cAMP response enhancer element (CRE) of the promoter [10]. The nuclear protein, CREB, is expressed ubiquitously, and it is suggested to regulate more than 4000 genes in several tissues, including the brain. Its activation leads to increased neuronal regeneration, neuronal survival, and enhances several neuronal processes [11]. CREB regulates the expression of anti-apoptotic and pro-apoptotic members of the Bcl-2 protein family [12].

We have investigated the role of CREB transcription factor overexpression in ethanol-induced ER stress and apoptosis in PC12 cells, a widely used model system to study various neuronal processes. In our previous experiments on PC12 cells, we have shown that various stressors such as serum starvation, anisomycin, and nitrosative agents induce apoptosis via caspase-dependent PKR activation, eIF2α phosphorylation, and intrinsic and extrinsic pathways [13,14]. These results highlight the relevance of this model for studying stress-related signaling and support the current investigation of the role of CREB in ethanol-induced ER stress.

## 2. Materials and Methods

### 2.1. Cell Cultures

The wild-type PC12 (wtPC12) rat pheochromocytoma cell line was isolated from an adrenal medulla tumor [15], and it was a gift from G. M. Cooper. Cells were cultured in Dulbecco’s Modified Eagle’s Medium (Sigma-Aldrich Chemical Co., St. Louis, MO, USA) containing 4500 mg/L glucose, 4 mM L-glutamine, and 110 mg/l sodium pyruvate, at 37 °C and 5% CO_2_. The medium was supplemented with 10% horse serum and 5% fetal bovine serum (FBS) (Gibco, Carlsbad, CA, USA). PC12 cells overexpressing CREB (PC12-CREB, established by M. Pap, see Balogh et al., 2014) [16] were cultured in the same medium containing 200 µg/mL geneticin (G418, Merck, Darmstadt, Germany). Both cell lines were treated with absolute ethanol (Sigma-Aldrich Chemical Co., St. Louis, MO, USA) at a concentration of 4 mg/mL for 24 and 48 h.

### 2.2. Reverse Transcription Quantitative PCR (RT-qPCR)

10^6^ cells were seeded onto 100 mm plates, harvested the next day and lysed in 200 µL TRIzol Reagent (Invitrogen, Waltham, MA, USA). Total RNA was isolated by Direct-zol RNA Miniprep Kit (Zymo Research) according to the manufacturer’s instructions. Sample concentrations were determined by a NanoDrop One Spectrophotometer (Thermo Scientific, Waltham, MA, USA), and 1000 ng RNA was reverse transcribed into complementary DNA by qPCRBIO cDNA Synthesis Kit (PCR Biosystems, London, UK) according to the manufacturer’s instructions. The following cDNA synthesis program was used: 25 °C, 5 min 30 s, 42 °C 55 min, 48 °C 5 min, and 80 °C 5 min. 1 µg cDNA and 10 pM/µL primers were used to perform the qPCR reaction by a CFX RT-PCR thermocycler (BioRad, Hercules, CA, USA). The program was as follows: initial denaturation 95 °C 3 min, 40 cycles of 95 °C 10 s, 55 °C 30 s, 72 °C 1 min. All samples were amplified in duplicate, and relative gene expression was analyzed using CFX Maestro Software (BioRad), and TATA-box binding Protein (TBP) was used as endogenous control. PrimerQuest software (version 2.2.3), obtained from the IDT website (Integrated DNA Technologies, Coralville, IA, USA), was used to design primer pairs. To detect the endogenous CREB expression, the following primer pairs were used: forward primer 5′-GACTGGCTTGGCCACAA-3′, reverse primer 5′-TGTAGTTGCTTTCAGGCAGTT-3′. To investigate exogenous CREB expression, we designed a primer sequence found only in the plasmid vector (forward primer 5′-GACTGGCTTGGCCACAA-3′, reverse primer 5′-GGCATAGATACCTGGGCTAATG-3′).

### 2.3. Cell Viability Assay

For the detection of cell viability ATP assay (Promega CellTiter-Glo^®^ 2.0 Cell Viability Assay, Madison, WI, USA) was used. 2000 PC12 and 1000 PC12-CREB cells per well were plated on a white, flat-bottom 96-well-plate (Greiner, Kremsmünster, Austria) coated with poly-L-lysine. The next day, the cells were treated with ethanol at 4 mg/mL concentration for 48 h, and for 24 h the next day. Measurements were performed according to the manufacturer’s instructions. Luminescence was detected by FLUOstar-OPTIMA V2.20 (BMG Labtech, Offenburg, Germany) luminometer. Four independent experiments with triplicate samples were performed and statistically analyzed.

### 2.4. Apoptosis Assay (Nuclear Morphology by Hoechst Staining)

10^5^ cells were plated in 24-well plates coated with poly-L-lysine (Sigma, St. Louis, MO, USA). The next day, cells were treated with 4 mg/mL ethanol for 48 h, and for 24 h the following day. After treatment, cells were fixed with 4% paraformaldehyde in 1 × PBS. The cell nuclei were stained with Hoechst 33342 (Sigma-Aldrich Chemical Co., St. Louis, MO, USA) fluorescent DNA dye dissolved in 1 × PBS at a concentration of 0.5 μg/mL. Samples were mounted with Vectashield (Vector Laboratories, Burlingame, CA, USA) anti-fading mounting medium. 200 cells/sample were counted in randomly chosen view fields (blinded to the experimental conditions) to determine the ratio of apoptotic nuclei using an Olympus BX61 fluorescence microscope (Olympus, Center Valley, PA, USA). The characteristic condensed and fragmented nuclear morphology was considered and counted as apoptotic nuclei. Representative fluorescence microscopy images illustrating the nuclear morphology of control and ethanol-treated wtPC12 cells are provided in the Supplementary Materials (Figure S2). All samples were analyzed in duplicate, and experiments were performed and analyzed independently four times.

### 2.5. Western Blotting Assay

5 × 10^6^ cells were plated onto 100 mm plates and treated with 4 mg/mL ethanol the next day for 24 and 48 h. After treatment, cells were harvested and lysed with M-Per Protein Extraction Buffer (Thermo Scientific, Waltham, MA, USA) supplemented with phosphatase inhibitor cocktail and protease inhibitor (Thermo Scientific, Waltham, MA, USA). Lysates were boiled for 5 min, and 40 µg protein was electrophoresed on 12% SDS-polyacrylamide gel. After electrophoresis, proteins were transferred onto polyvinylidene difluoride membrane (PVDF, Thermo Fisher Scientific, Rockford, IL, USA), and blots were blocked with 5% non-fat milk in 1 × PBS for 1 h. After blocking, the membranes were treated with the appropriate primary antibodies according to the manufacturer’s protocols. The following primary antibodies were used in 1:1000 dilution: anti-BiP, anti-CHOP, anti-ATF-6, anti-P-JNK, anti-P-p38, anti-P-p53, anti-Bim, anti-Puma, anti-cleaved Caspase-3, anti-Mcl-1, anti-P-Bad, anti-CREB, anti-β-Actin (Cell Signaling, Danvers, MA, USA). After washing the blots five times in 1 × TBS-T 0.1% Tween-20 buffer for 5 min, horseradish peroxidase-conjugated secondary antibodies (anti-rabbit, anti-mouse in 1:2000 dilution, Cell Signaling Technology, Danvers, MA, USA) were used to detect bound primary antibodies. The results were visualized by G-Box gel documentation system (Syngene, Synoptics, Cambridge, UK) after washing the membranes ten times for 5 min. At least three independent experiments were performed. Band intensity was quantified using ImageJ software 1.54g (NIH, Bethesda, MD, USA), and target protein expression levels were normalized to the control samples, respectively. Full-length, uncropped blots corresponding to the cropped images shown here are presented in the Supplementary Materials (Figure S1).

### 2.6. Statistical Analysis

Statistical analysis was conducted with the statistical analysis program PAST v2.17c, and the significance of the data was analyzed by the Kruskal–Wallis test. The normal distribution was determined by the Shapiro–Wilk test. Data are presented as mean  ±  standard deviation. The number of biological and technical replicates for each experiment is indicated in the Figure legends. *p* < 0.05 value was considered statistically significant.

## 3. Results

### 3.1. Quantification of Exogenous CREB Expression in Stable Transfected PC12-CREB Cells

**Figure 1:**
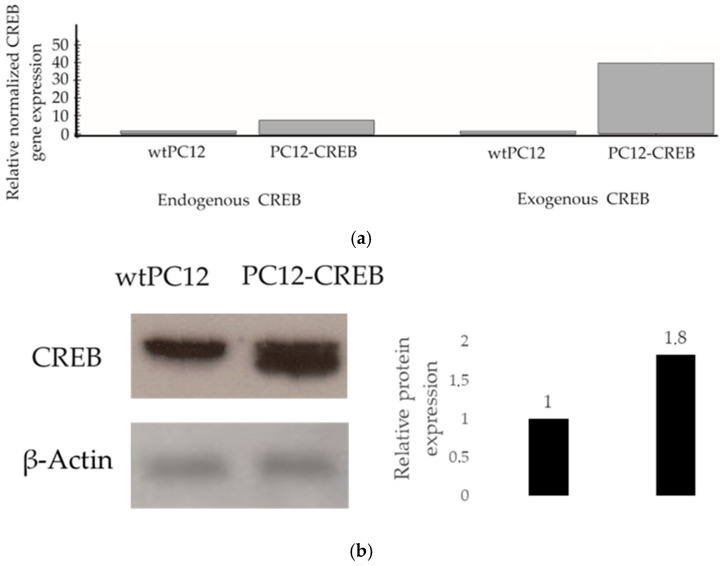
RT-qPCR and Western blot analysis of endogenous and exogenous CREB expression in wtPC12 and PC12-CREB cells. (**a**) Relative mRNA expression levels of CREB were determined by RT-qPCR. (**b**) CREB protein levels were analyzed by Western blot. The experiments were performed once with the primary aim of verifying stable overexpression of CREB in the PC12-CREB cell line. Therefore, no statistical analysis was applied. β-Actin was used as a loading control. The bar graph next to the blots shows densitometric quantification of protein bands normalized to β-Actin, based on ImageJ analysis. Details of the experiments are described in the Section 2. Full-length, uncropped blots corresponding to the cropped images shown here are presented in the Supplementary Materials (Figure S1).

Reverse transcription real-time PCR (RT-qPCR) was used to examine the expression of endogenous CREB and exogenous CREB to demonstrate that stable transfected PC12-CREB cells overexpress CREB mRNA (Figure 1a). In wild-type PC12 (wtPC12) cells, neither endogenous CREB nor exogenous CREB mRNA showed elevated levels. In PC12 cells transfected with CREB-expressing plasmids (PC12-CREB), endogenous CREB mRNA expression showed low levels similar to wtPC12 cells. The level of exogenous CREB in transfected PC12-CREB cells showed a significant increase of approximately 40-fold compared to wtPC12 cells. In conclusion, PC12-CREB cells overexpress CREB transcription factor mRNA, whereas wtPC12 cells do not. The expression of CREB protein was also detected by Western blot analysis in both cell lines (Figure 1b). PC12-CREB cells express CREB protein at a higher amount (fold change in protein level: 1.8) compared to the wtPC12 cells.

### 3.2. Effect of Ethanol on the Viability of wtPC12 and PC12-CREB Cells

**Figure 2:**
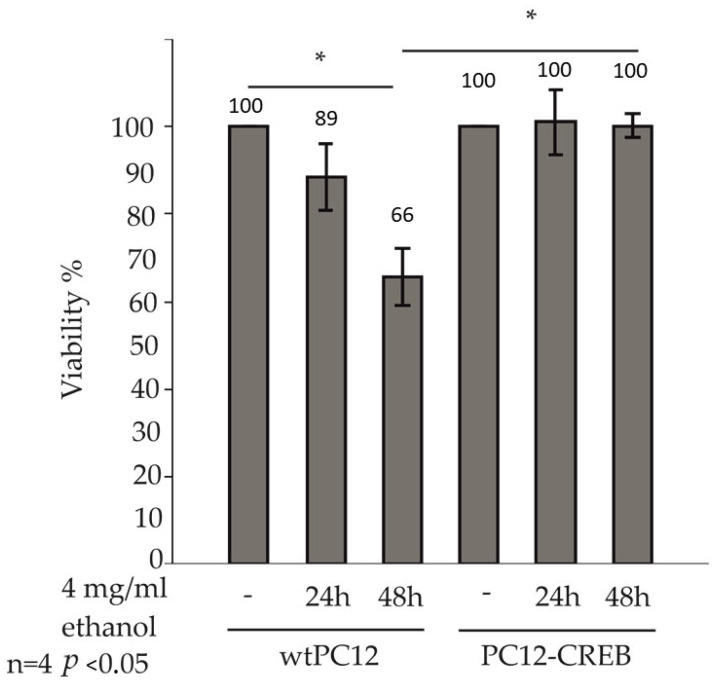
Effect of ethanol treatment on cell viability of wtPC12 and PC12-CREB cells. The cells were treated with 4 mg/mL ethanol for 24 or 48 h, and the viability of the cells was determined by ATP assay. Four independent experiments with duplicate samples were performed and statistically analyzed by the Kruskal–Wallis test. The data for ethanol-treated samples were normalized to the untreated control, which was set to 100% as a reference. Therefore, standard deviation values are not displayed for control samples. Data are presented as mean  ±  standard deviation. Statistically significant differences (*p* < 0.05) are marked with an asterisk. Details of the experiments are described in the Section 2.

The ATP assay is a widely used and accepted method to study cell viability. This method was used to determine whether alcohol has a differential effect on the viability of wtPC12 and CREB-transfected PC12 cells (Figure 2).

In our treatments, 4 mg/mL ethanol concentration was used, which was determined according to the scoring in Physiological effects of various blood alcohol levels [17], where a blood alcohol concentration of 0.4% causes stupor, coma, respiratory depression, hypothermia, and is highly toxic to cells.

The viability of treated cells was normalized to the viability of untreated control cells, respectively. Viability of wtPC12 cells decreased after 24 h of ethanol treatment compared to untreated control cells. 48 h of ethanol treatment caused a significant decrease in ATP concentration in wild-type PC12 cells. ATP concentration of wtPC12 cells decreased to 68%, which represents a significantly lower viability compared to untreated control cells. PC12-CREB cell viability did not change after 24 h and 48 h of treatment compared to control cells. The viability of PC12-CREB cells was significantly higher than that of wtPC12 cells after 48 h of treatment.

### 3.3. Effect of Ethanol on Apoptosis in Wild-Type and CREB-Overexpressing Cells

**Figure 3:**
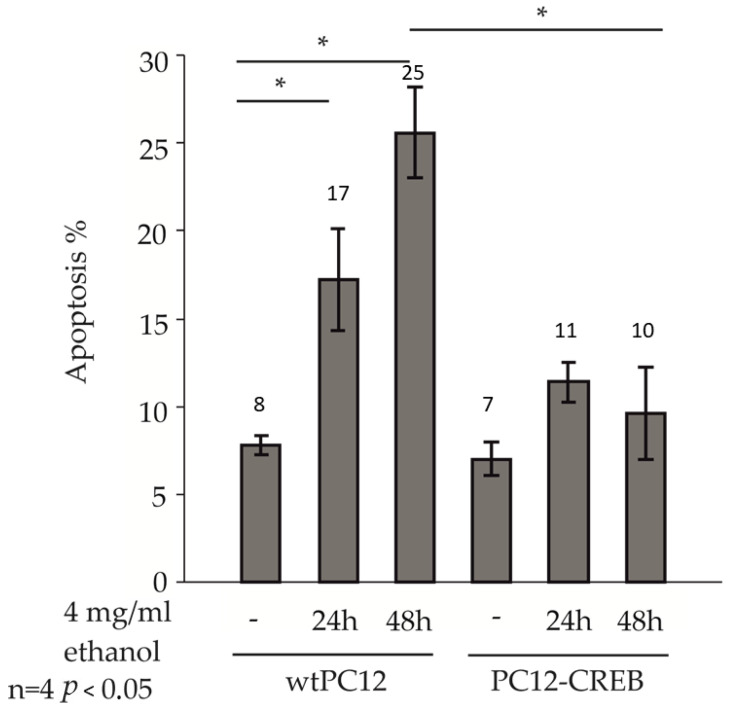
Effect of ethanol treatment on apoptosis of wtPC12 and PC12-CREB cells. The cells were treated with 4 mg/mL ethanol for 24 or 48 h, fixed, and the cell nuclei were stained with Hoechst 33,342 dye. The morphology of normal and apoptotic cell nuclei was detected in a fluorescence microscope, and the percentage of apoptotic cells was determined. Representative fluorescence microscopy images illustrating the nuclear morphology of control and ethanol-treated wtPC12 cells are provided in the Supplementary Materials (Figure S2). Four independent experiments with duplicate samples were performed and statistically analyzed by the Kruskal–Wallis test. Data are presented as mean  ±  standard deviation. Statistically significant differences (*p* < 0.05) are marked with an asterisk. Details of the experiments are described in the Section 2.

Control and 4 mg/mL ethanol-treated wtPC12 and PC12-CREB cells for 24 and 48 h, were observed in a fluorescence microscope after staining with Hoechst dye (Figure 3). The percentage of apoptotic nuclei was determined based on the characteristic fragmented nuclear morphology by counting at least 200 cells/sample in randomly chosen view fields using a fluorescence microscope. (Representative images are shown in Supplementary Figure S2).

Untreated cells in both cell lines showed approximately 7–8% apoptotic levels. After 24 and 48 h of ethanol treatment, the percentage of apoptotic cells increased in wtPC12 cells. The 24 h ethanol treatment evoked a significantly higher rate of apoptosis (17%) compared to untreated wtPC12 cells. The prolonged treatment induced apoptosis in approximately 25% of the cells, which was significantly higher than in the control cells.

In the stable transfected PC12-CREB cell line, ethanol treatment increased the percentage of apoptotic cells (10–11%) compared to control cells, but was not as pronounced as in the wild-type cell line. The levels of apoptotic cells were not significantly different after 24 or 48 h of ethanol treatment. The 48 h ethanol treatment caused a significantly higher rate of apoptosis in wtPC12 cells than in PC12-CREB cells.

### 3.4. Effects of Ethanol Treatment on ER Stress and Apoptosis

**Figure 4:**
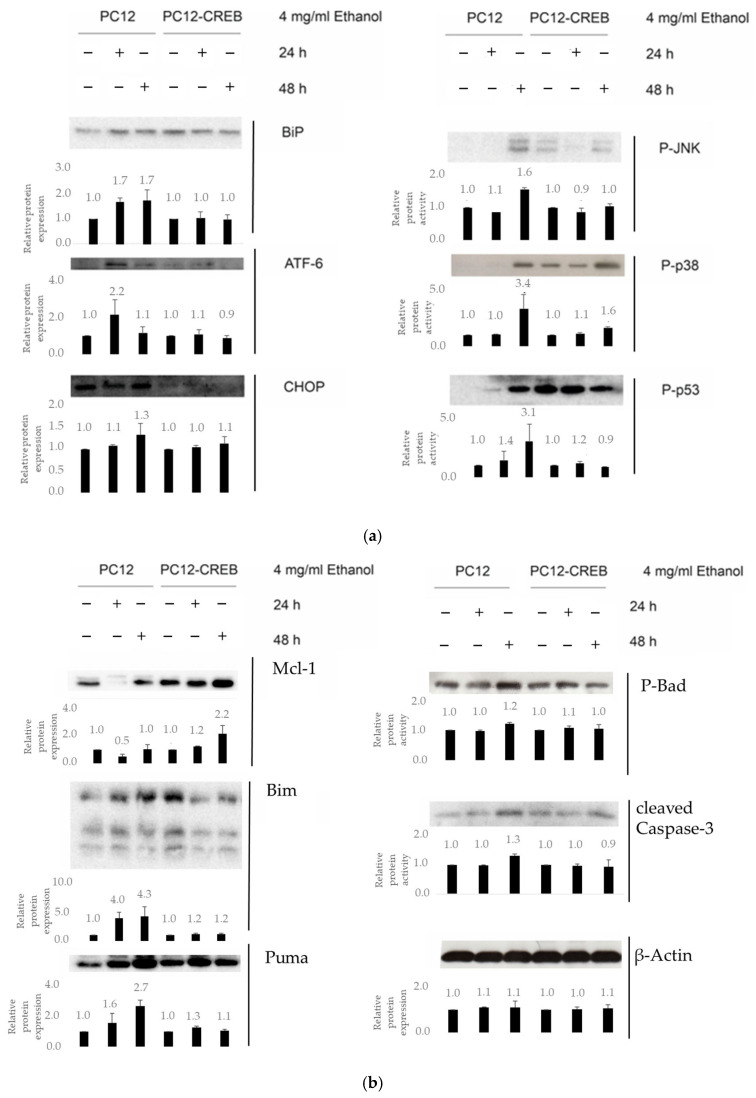
Representative Western blot analysis of proteins involved in the regulation of ER stress (**a**) and apoptosis (**b**) in wtPC12 and PC12-CREB cells treated with ethanol for 24 or 48 h. β-Actin was used as a loading control. Bar graphs below the blots show densitometric quantification of protein bands normalized to the control samples, respectively, based on ImageJ analysis. Data are presented as mean  ±  standard deviation. At least three independent experiments were performed. Details of the experiments are described in the Section 2. Full-length, uncropped blots corresponding to the cropped images shown here are presented in the Supplementary Materials (Figure S1).

Dissociation of BiP protein from the ER transmembrane sensor proteins is a key step in the ER stress. BiP levels were increased in wtPC12 cells after 24 (fold change in protein level: 1.7 ± 0.14) and 48 h (fold change in protein level: 1.7 ± 0.41) of ethanol treatment compared to untreated cells. In PC12-CREB cells, BiP expression was decreased by ethanol treatment compared to control samples. ATF6 sensor protein expression was increased after 24 h of alcohol treatment in both wtPC12 (fold change in protein level: 2.2 ± 0.8) and PC12-CREB (fold change in protein level: 1.1 ± 0.29) cells, but to a much lower extent in the latter CREB-overexpressing cell line. The apoptosis-regulating transcription factor CHOP is upregulated following ethanol treatment in the wild-type cells compared to control cells (fold change in protein level after 48 h treatment: 1.3 ± 0.26), whereas its expression remains unchanged in stable CREB-transfected cells. Both p38 MAPK and JNK, consistent with their roles, play important functions in mediating ER stress. Both kinases were activated after 48 h of ethanol treatment in the wtPC12 cells. JNK underwent a strong phosphorylation in wild-type cells after 48 h of treatment (fold change in protein level: 1.6 ± 0.05), while the rate of phosphorylation in CREB-transfected cells showed significantly attenuated levels (fold change in protein level: 1.0 ± 0.06). 48 h of ethanol treatment caused an increased p53 phosphorylation in wtPC12 and PC12-CREB cells compared to the untreated cells, but the elevation was higher in the wild-type cells (fold change in protein level: 3.1 ± 1.5) (Figure 4a). The ethanol treatment also influenced the expression of apoptosis regulator Bcl-2 family member proteins. Anti-apoptotic Mcl-1 protein did not show an elevation after ethanol treatment in wtPC12 cells (fold change in protein level after 48 h treatment: 1.0 ± 0.3), while the 48 h ethanol treatment induced the expression of this protein in PC12-CREB cells (fold change in protein level after 48 h treatment: 2.2 ± 0.6). Bim is a BH3-only domain pro-apoptotic protein, which is involved in the inhibition of the anti-apoptotic proteins. In the wtPC12 cells, the 24 h ethanol treatment caused an elevation in the Bim level (fold change in protein level: 4.0 ± 1.02), while the 48 h treatment had a stronger effect on Bim expression (fold change in protein level: 4.3 ± 1.68). It is evident that CREB overexpression increases the Bim level in the PC12-CREB cells, but the ethanol treatment decreases the Bim expression (fold change in protein level: 1.2 ± 0.18). Puma, a pro-apoptotic protein showed a higher expression in the wild-type cells after 24 h treatment (fold change in protein level: 1.6 ± 0.63), and the elevation was more pronounced after 48 h (fold change in protein level: 2.7 ± 0.4). The PC12-CREB cells also had an elevated Puma protein level compared to the control (fold change in protein level after 24 h treatment: 1.3 ± 0.1), but not as prominent as in the wild-type cells. Bad pro-apoptotic protein underwent phosphorylation after 48 h ethanol treatment in the wild-type cells (fold change in protein level: 1.2 ± 0.05), but in the stable transfected cells, the phosphorylation status was not changed compared to the untreated cells (fold change in protein level: 1.0 ± 0.13). Caspase-3 is an important executor of apoptosis [18], it showed an elevated level of the cleaved form in both ethanol-treated cell lines compared to the untreated cells, but in the wtPC12 cells, the elevation was more prominent (fold change in protein level after 48 h treatment: 1.30 ± 0.05) (Figure 4b).

## 4. Discussion

Chronic alcohol consumption impairs behavioral and cognitive functions in the brain, and at the cellular level induces oxidative stress, leading to protein misfolding in the ER and activation of the UPR. Compulsive alcohol intake also affects cellular stress responses in the cells that affect the cAMP signaling pathway, which is closely linked to neuronal survival [19].

CREB is a leucine zipper transcription factor expressed ubiquitously in various tissues, including the brain, and is known to regulate genes involved in neuronal survival, plasticity, and apoptosis through binding to CRE [20]. The effects of ethanol on CREB activation are controversial. Some studies report that ethanol exposure increases CREB phosphorylation, thereby inducing the expression of target genes [19], while others show that it reduces intracellular cAMP levels and CREB activity, potentially by inhibiting adenylate cyclase or stimulating phosphodiesterases [21].

The PC12 cell line is a well-established neuronal model to study differentiation, apoptosis, and ER stress [13,14,15]. We generated a stable PC12 line overexpressing CREB to examine its role in ethanol-induced ER stress and apoptosis compared with wild-type cells.

Ethanol concentrations of 0.4% or higher are widely used both in vivo and in vitro to induce ER stress and apoptosis. For instance, Tiwari et al. administered ethanol (5 g/kg, 12% *v*/*v*) to rat pups postnatally and detected impaired memory performance and an increase in the expression of proteins involved in stress signaling and apoptosis (e.g., TNF-α, IL-1β, TGF-β, NFκB, and caspase 3) [22]. In a cell culture study 0.4% ethanol significantly reduced cell viability and upregulated multiple ER stress markers (e.g., BiP/GRP78, ATF6) and induced apoptosis, demonstrated by an increase in cleaved caspase 3 level in mouse pancreatic acinar 266–6 cells [23]. Sushma et al. reported that 0.5–4% ethanol concentrations caused a significant decrease in cell viability at 48 h of exposure period, induced significant upregulation of pro-apoptotic marker proteins and ER stress sensor proteins (e.g., IRE1, eIF2α, and CHOP), and the downregulation of Bip protein in U-87 (Human glioblastoma) and HMC-3 (Human microglia) cell lines [24]. In PC12 cells, ~0.46% ethanol enhanced apoptosis via caspase-3/-9 activation and cytochrome c release [25], and promoted lipid peroxidation in a dose-dependent manner [26].

Based on our preliminary dose–response experiments, we selected 4 mg/mL (0.4% *v*/*v*) ethanol concentration for the present study, because it consistently revealed reproducible differences between the different cell lines. Our preliminary time-course experiments indicated that 24 h and 48 h long ethanol treatments were the most informative and reproducible to detect dynamic changes in CREB-related stress and apoptotic signaling, which is why we selected them for further detailed analysis.

Having established the optimal ethanol concentration and time points, we next assessed the viability of wtPC12 and PC12-CREB cells by ATP assays. Ethanol treatment significantly decreased the viability of wtPC12 cells, while CREB overexpression prevented this effect. We next examined whether apoptosis induced by ethanol treatment could be blocked in PC12-CREB cells. Overexpression of CREB protected cells from apoptosis and showed significantly lower apoptotic cell numbers in PC12-CREB cells.

Subsequently, we investigated the expression levels of markers indicative of ER stress and apoptosis. Protein misfolding in the ER leads to the dissociation of BiP from ATF6, facilitating the translocation of ATF6 to the nucleus where it activates the expression of ER stress response genes. Increased BiP expression enhances ER protein-folding capacity and mitigates ER stress [27]. Short-term ethanol treatment increased ATF6 levels and BiP expression in wtPC12 cells, indicating ER stress activation. In contrast, CREB-overexpressing cells displayed attenuated ATF6 and BiP levels, suggesting that CREB counteracts ER stress induction. Since ATF6 promotes antioxidant effects by inducing the expression of antioxidant proteins that eliminate ROS and reduce ER stress, its suppression may reflect an overall reduction in ER stress load [27].

CHOP, a pro-apoptotic transcription factor downstream of all three UPR branches, was strongly upregulated following ethanol exposure, accompanied by JNK and p38 MAPK activation—two major stress kinases linked to ER stress and apoptosis [6,28]. CREB overexpression attenuated CHOP induction and JNK phosphorylation, with a less pronounced effect on p38 MAPK. These data suggest that CREB modulates ER stress not only by influencing chaperone gene expression but also by suppressing pro-apoptotic signaling cascades.

Consistent with these findings, cells overexpressing CREB exhibited reduced levels of Puma and Bim, BH3-only pro-apoptotic members of the Bcl-2 family, while sustaining elevated levels of Mcl-1, a critical anti-apoptotic protein. These findings support the known role of CREB in promoting Mcl-1 transcription and inhibiting apoptosis by regulating Bcl-2 family members [12,29]. Indeed, the increased cell survival and reduced caspase-3 activation observed in CREB-overexpressing cells confirm its neuroprotective role under ethanol-induced stress.

These results are consistent with our previous findings in PC12 cells, where various stressors, including serum deprivation, anisomycin, and cytotoxic agents, induced apoptosis via caspase-dependent cleavage of protein kinase R (PKR), activation of eIF2α, and modulation of p53-related pathways [13,14]. In these studies, impaired p53 function reduced stress-induced apoptosis, underscoring the importance of transcription factors in the regulation of neuronal cell fate under stress. Together with our current results, these findings highlight the CREB pathway as a key modulator of neuronal stress responses and a potential target for attenuating alcohol-induced neurotoxicity.

However, while our data strongly suggest that CREB overexpression confers a protective effect against ethanol-induced ER stress and apoptosis, the underlying transcriptional mechanisms remain inadequately characterized. We did not perform CRE reporter assays, CREB knockdown experiments, or specific pathway inhibitors to directly confirm CREB transcriptional activity under ethanol exposure. These experiments would provide further insight into how CREB regulates downstream targets in this context. Therefore, future studies should include CRE-luciferase reporter assays to evaluate CREB-driven gene transcription, as well as pharmacological inhibition or siRNA-mediated knockdown of specific downstream targets to further validate the neuroprotective mechanism of CREB. Importantly, these limitations do not alter our core conclusion that CREB overexpression reduces ER stress and apoptosis during ethanol exposure.

## 5. Conclusions

In this study, we show that the stable overexpression of CREB in PC12 cells attenuates ethanol-induced endoplasmic reticulum stress and apoptosis. Cells overexpressing CREB showed higher viability, reduced apoptotic cell numbers, and decreased activation of key ER stress and pro-apoptotic signaling molecules, including ATF6, BiP, CHOP, JNK and p38 MAPK, while maintaining elevated levels of the anti-apoptotic protein Mcl-1 and decreased expression of the pro-apoptotic proteins Puma and Bim. These results support the neuroprotective role of CREB under ethanol-induced stress conditions, providing further evidence for the involvement of CREB in regulating cellular stress responses.

Although the precise transcriptional mechanisms by which CREB mediates these protective effects remain to be fully elucidated, our results highlight CREB as a potential target for modulating neuronal resistance to alcohol-induced cellular stress. Future studies that directly assess CREB transcriptional activity, coupled with pathway inhibition and validation in additional neuronal models, will be essential to further elucidate the molecular mechanisms underlying its protective role.

## Data Availability

The original contributions presented in this study are included in the article/supplementary material. Further inquiries can be directed to the corresponding authors

## Supplementary Materials

The following supporting information can be downloaded at: https://www.mdpi.com/article/10.3390/biology14091277/s1, Figure S1: Full-length, uncropped Western blots; Figure S2: Nuclear morphology of control (a) and ethanol-treated wtPC12 cells (b).

## Abbreviations

The following abbreviations are used in this manuscript:

ATF4	activating transcription factor 4
ATF6	activating transcription factor 6
CHOP	C/EBP-homologous protein
CRE	cAMP response enhancer element
CREB	Cyclic AMP (cAMP)-response element-binding protein 1
eIF2α	eukaryotic translation initiation factor 2
ER	endoplasmic reticulum
ERAD	ER-associated degradation
FBS	fetal bovine serum
GRP78/BiP	glucose-regulated protein 78
IRE1	inositol-requiring enzyme 1
JNK	Jun N-terminal kinase
p38 MAPK	p38 mitogen-activated protein kinase
PC12-CREB	PC12 cells overexpressing CREB
PERK	protein kinase RNA-like ER kinase
PVDF	polyvinylidene difluoride membrane
ROS	reactive oxygen species
RT-qPCR	Reverse transcription real-time PCR
TBP	TATA-box binding Protein
UPR	Unfolded Protein Response
WHO	World Health Organisation
wtPC12	wild-type PC12, rat pheochromocytoma cell line
XBP1	X-box binding protein
