# Supplementary material for: Role of the Transcription Factor CREB in Ethanol-Induced Endoplasmic Reticulum Stress and Apoptosis in PC12 Cells
Source: Biology (Basel). 2025 Sep 16;14(9):1277. doi: 10.3390/biology14091277 (PMC12467229; doi:10.3390/biology14091277)
Supplement: Supplementary file 1 [file biology-14-01277-s001.zip › biology-3795769 Supplementary Figure S2.pdf]

Figure S2.

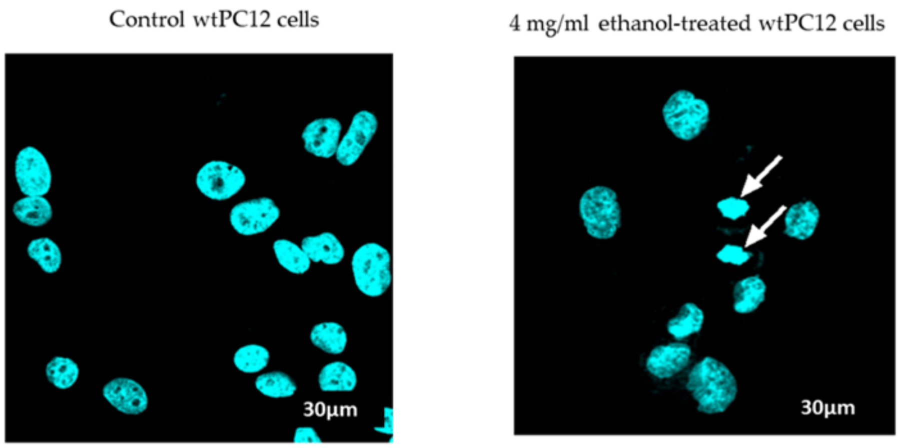

(a) **(b)**

Figure S2. Nuclear morphology of control (a) and ethanol-treated wtPC12 cells (b). Hoechst staining was performed as described in the "Materials and methods" section. The percentage of apoptotic nuclei (shown by white arrows) was determined by counting at least 200 cells/sample in randomly chosen view fields using an Olympus BX61 fluorescence microscope (Olympus, Center Valley, PA, USA). Four independent experiments were performed. Representative images are shown. The scale bar represents 30 µm.
