# Supplementary figures and images for: Role of the Transcription Factor CREB in Ethanol-Induced Endoplasmic Reticulum Stress and Apoptosis in PC12 Cells
Source: Biology (Basel). 2025 Sep 16;14(9):1277. doi: 10.3390/biology14091277 (PMC12467229; doi:10.3390/biology14091277)

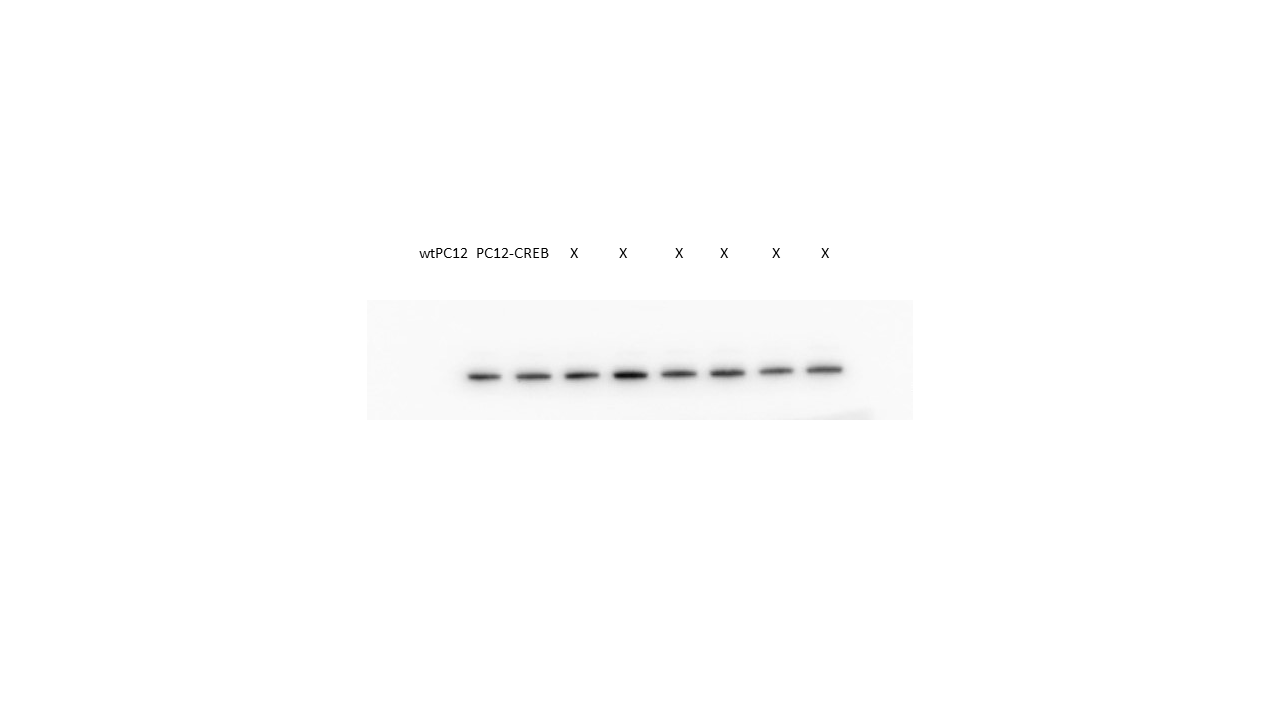

Supplement: Supplementary file 1 [file biology-14-01277-s001.zip › biology-3795769-Supplementary Figure S1/Figure_1b_ACTIN.tif]

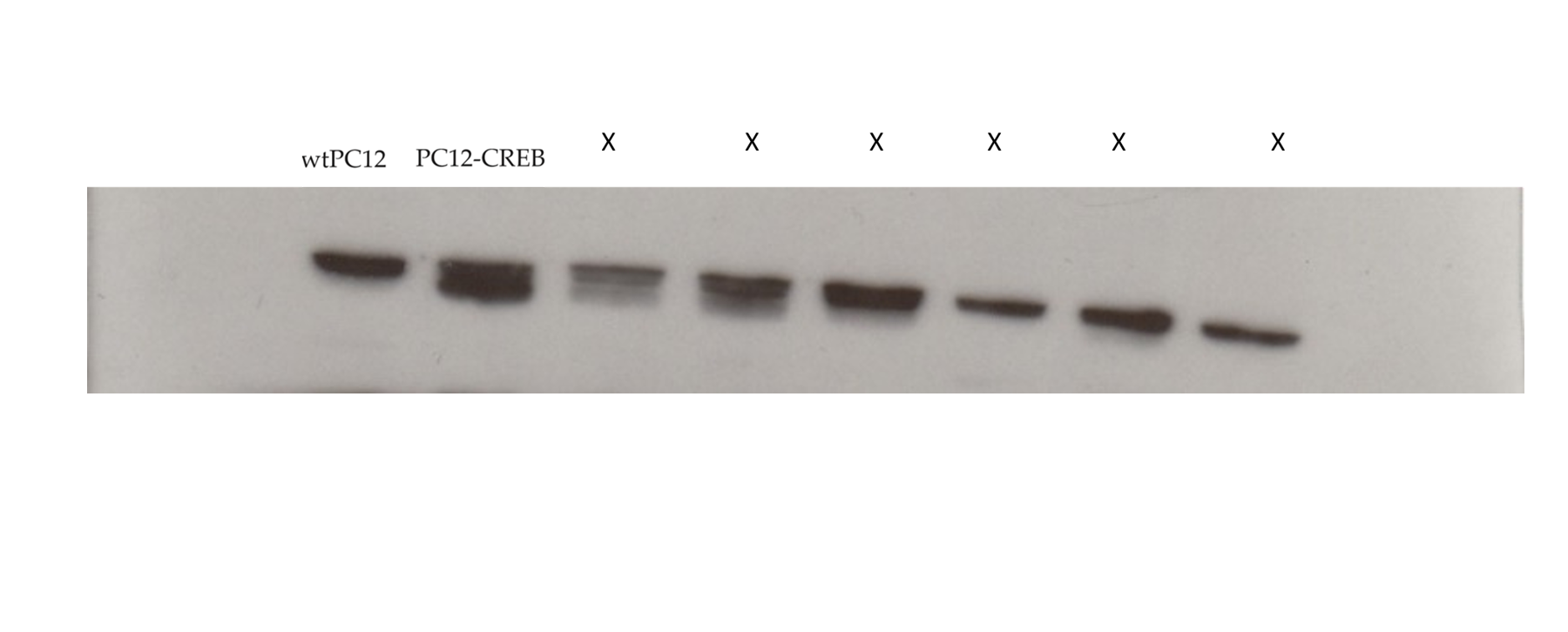

Supplement: Supplementary file 1 [file biology-14-01277-s001.zip › biology-3795769-Supplementary Figure S1/Figure_1b_CREB.tif]

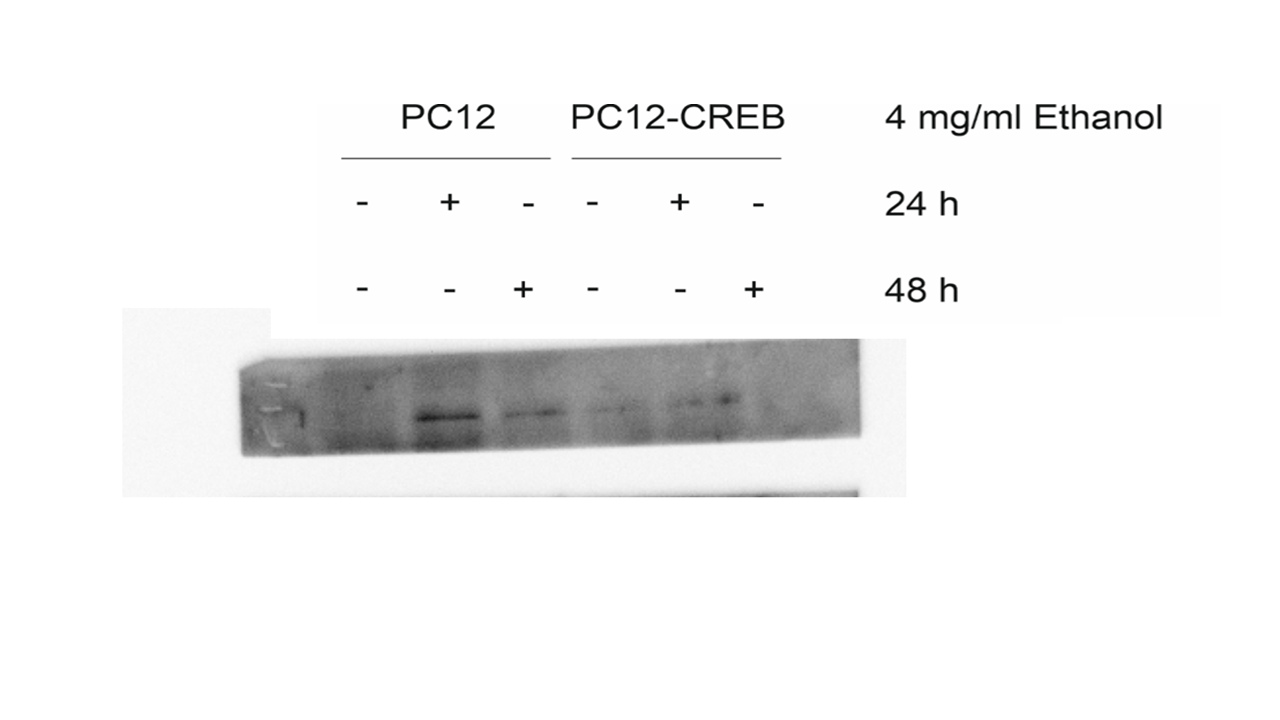

Supplement: Supplementary file 1 [file biology-14-01277-s001.zip › biology-3795769-Supplementary Figure S1/Figure_4a_ATF6.tif]

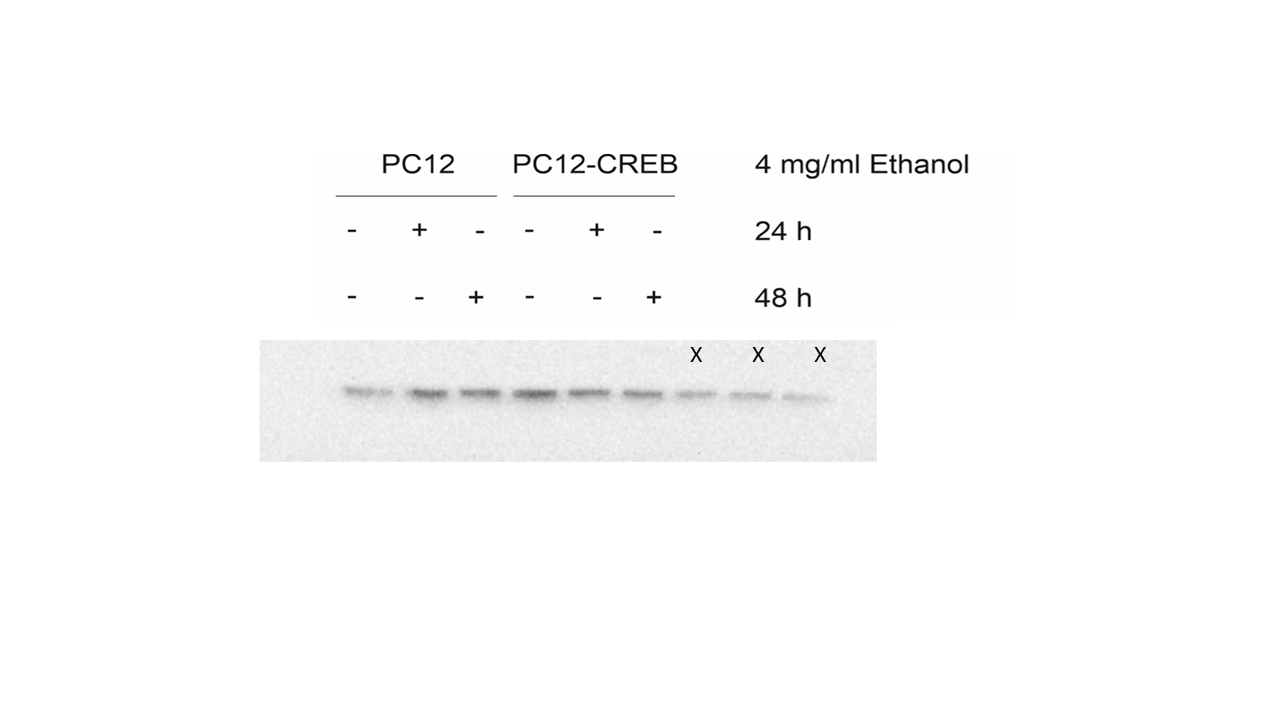

Supplement: Supplementary file 1 [file biology-14-01277-s001.zip › biology-3795769-Supplementary Figure S1/Figure_4a_BiP.tif]

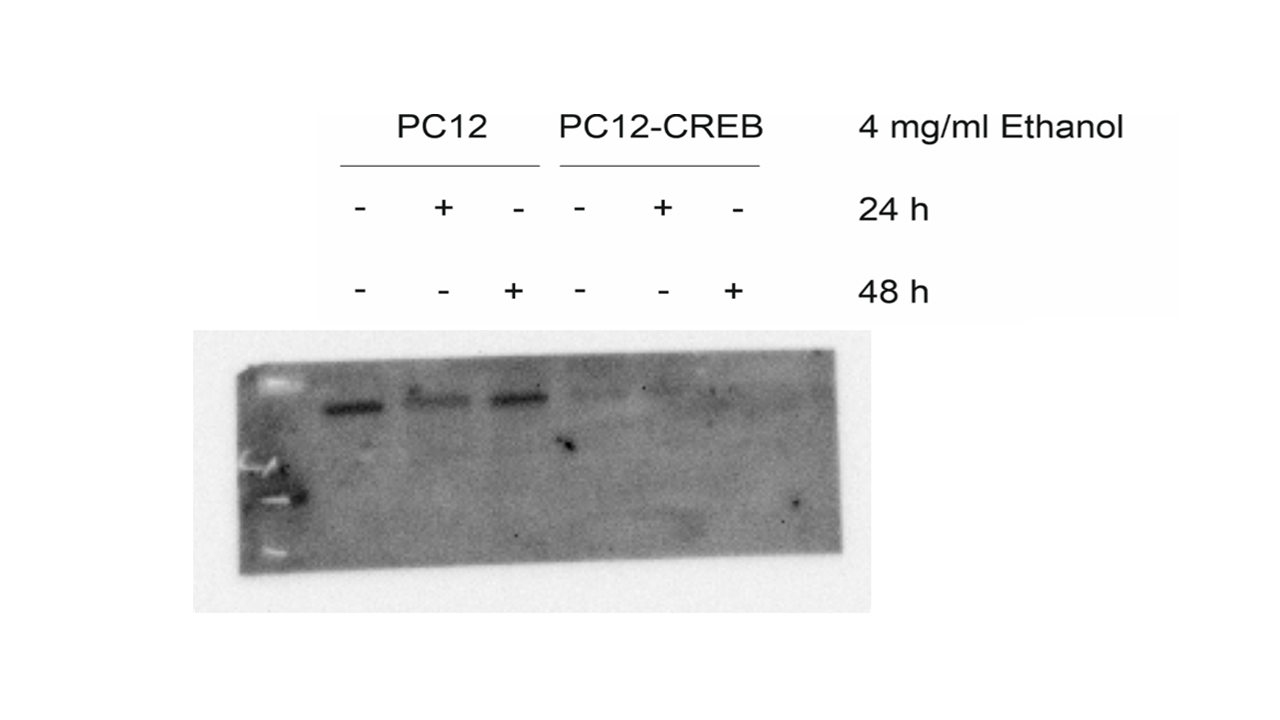

Supplement: Supplementary file 1 [file biology-14-01277-s001.zip › biology-3795769-Supplementary Figure S1/Figure_4a_CHOP.tif]

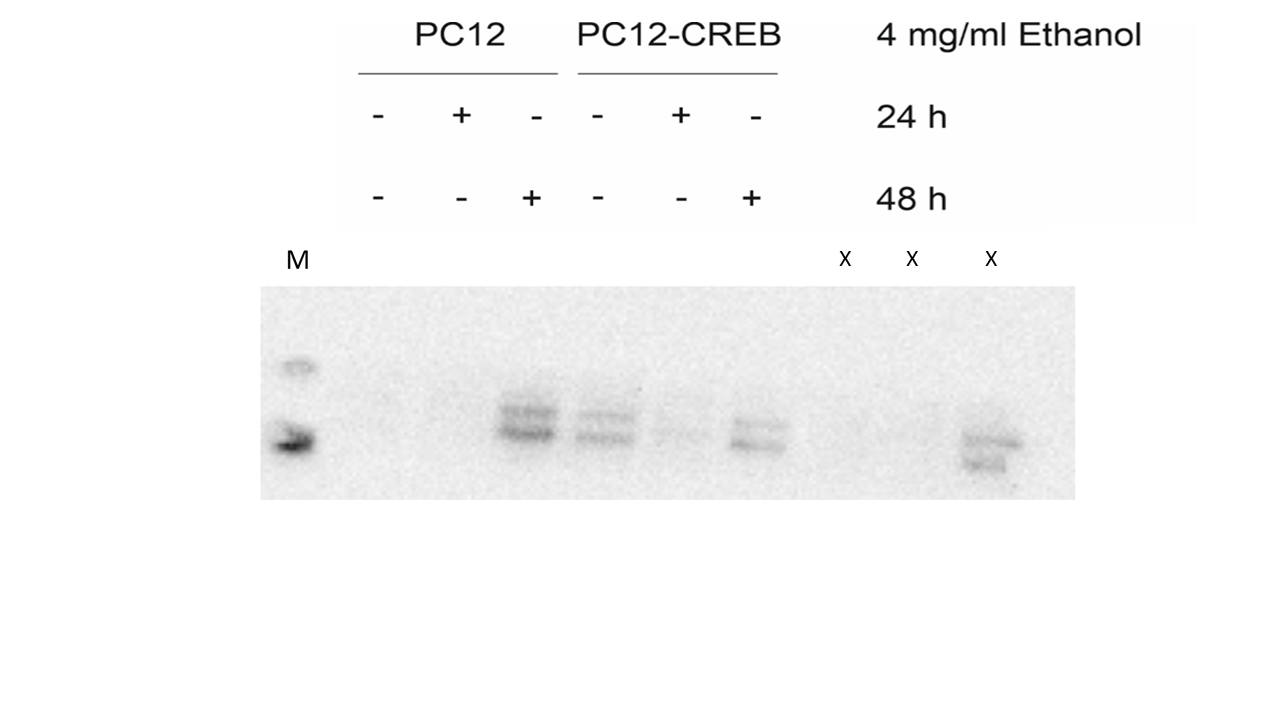

Supplement: Supplementary file 1 [file biology-14-01277-s001.zip › biology-3795769-Supplementary Figure S1/Figure_4a_P-JNK.tif]

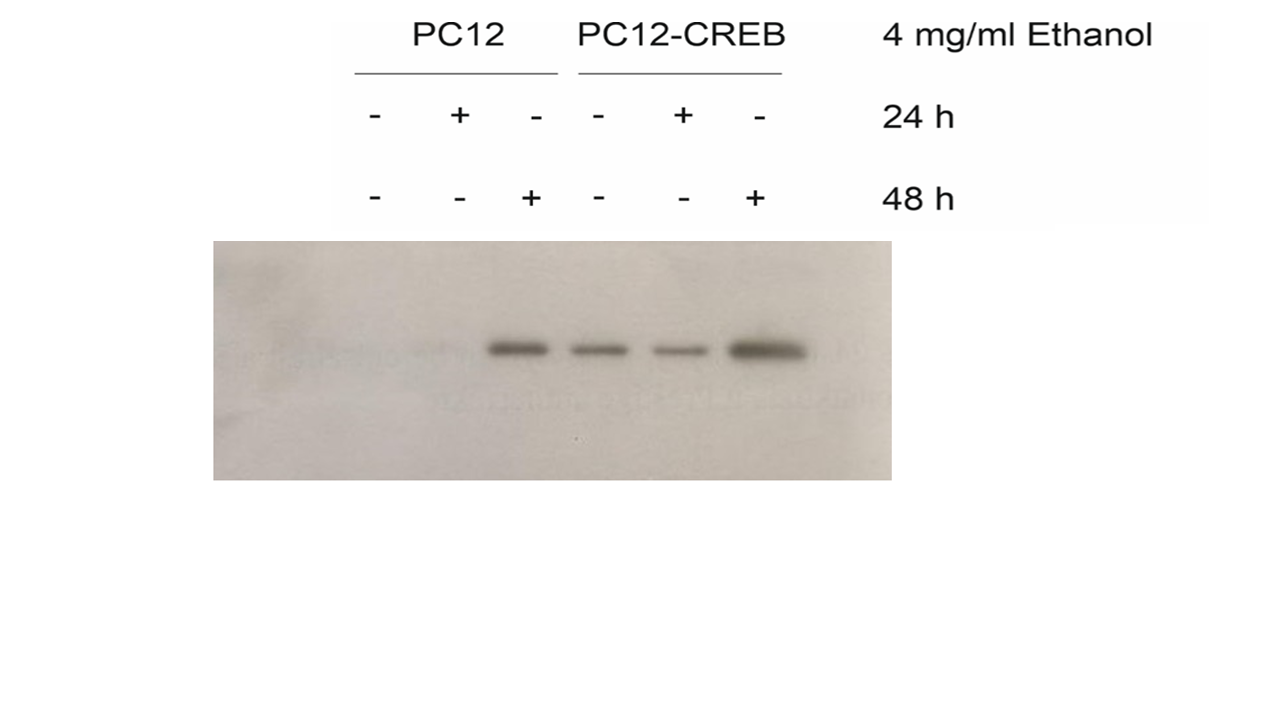

Supplement: Supplementary file 1 [file biology-14-01277-s001.zip › biology-3795769-Supplementary Figure S1/Figure_4a_P-p38.tif]

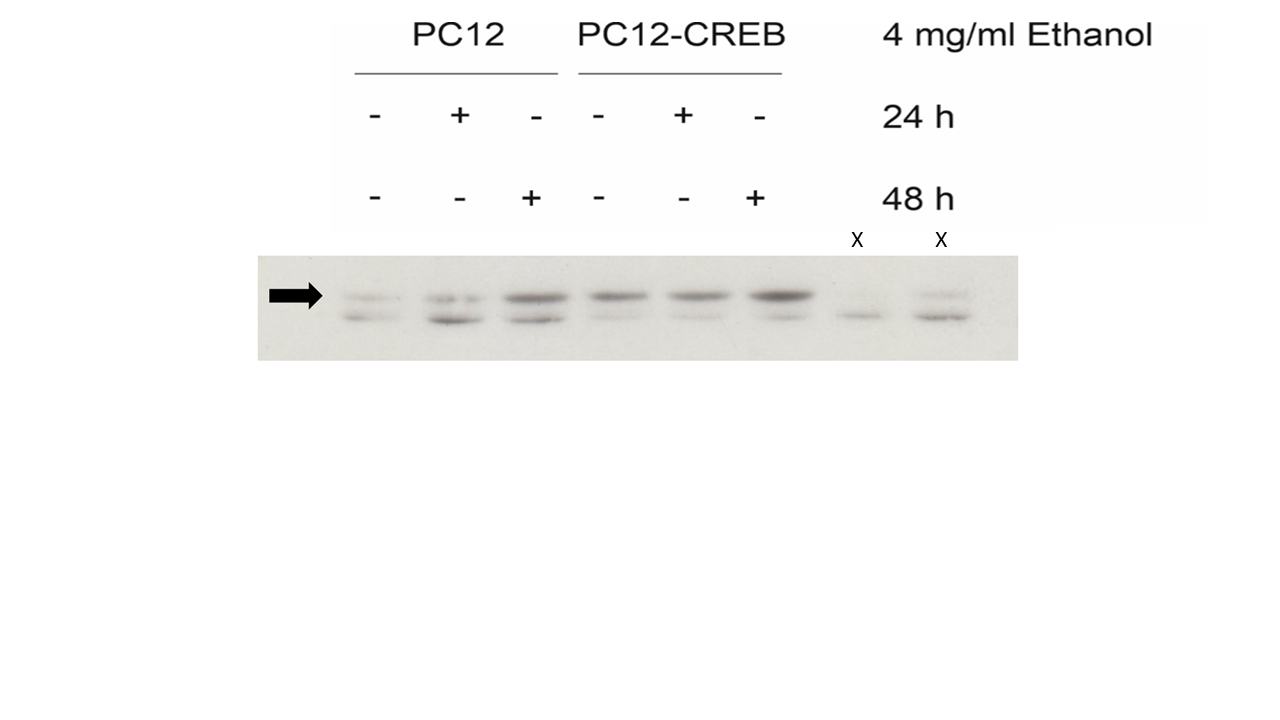

Supplement: Supplementary file 1 [file biology-14-01277-s001.zip › biology-3795769-Supplementary Figure S1/Figure_4a_P-p53.tif]

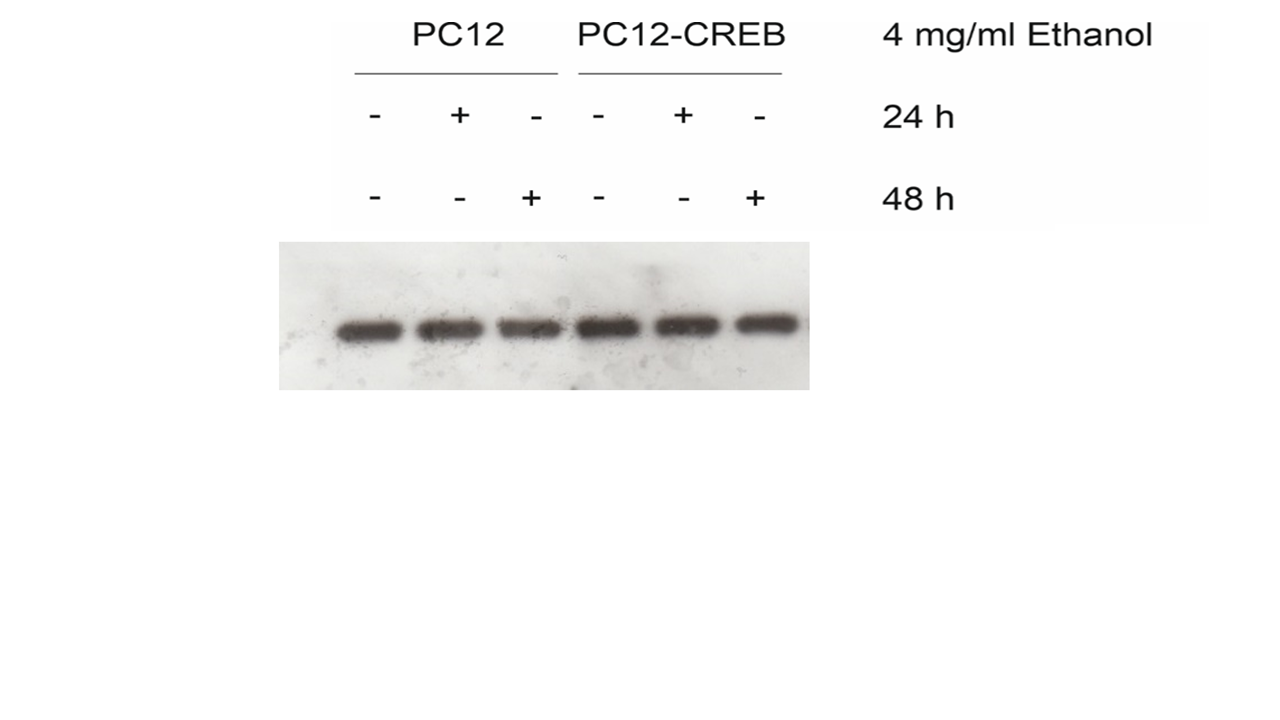

Supplement: Supplementary file 1 [file biology-14-01277-s001.zip › biology-3795769-Supplementary Figure S1/Figure_4b_Actin.tif]

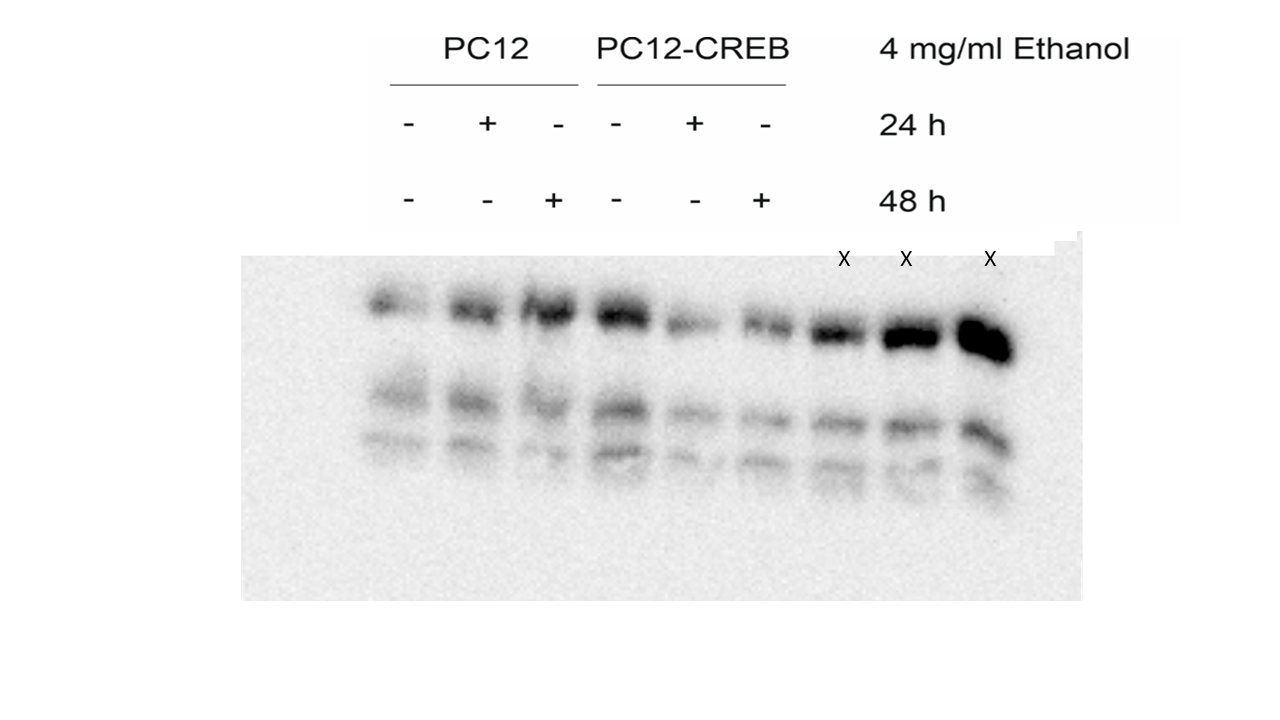

Supplement: Supplementary file 1 [file biology-14-01277-s001.zip › biology-3795769-Supplementary Figure S1/Figure_4b_Bim.tif]

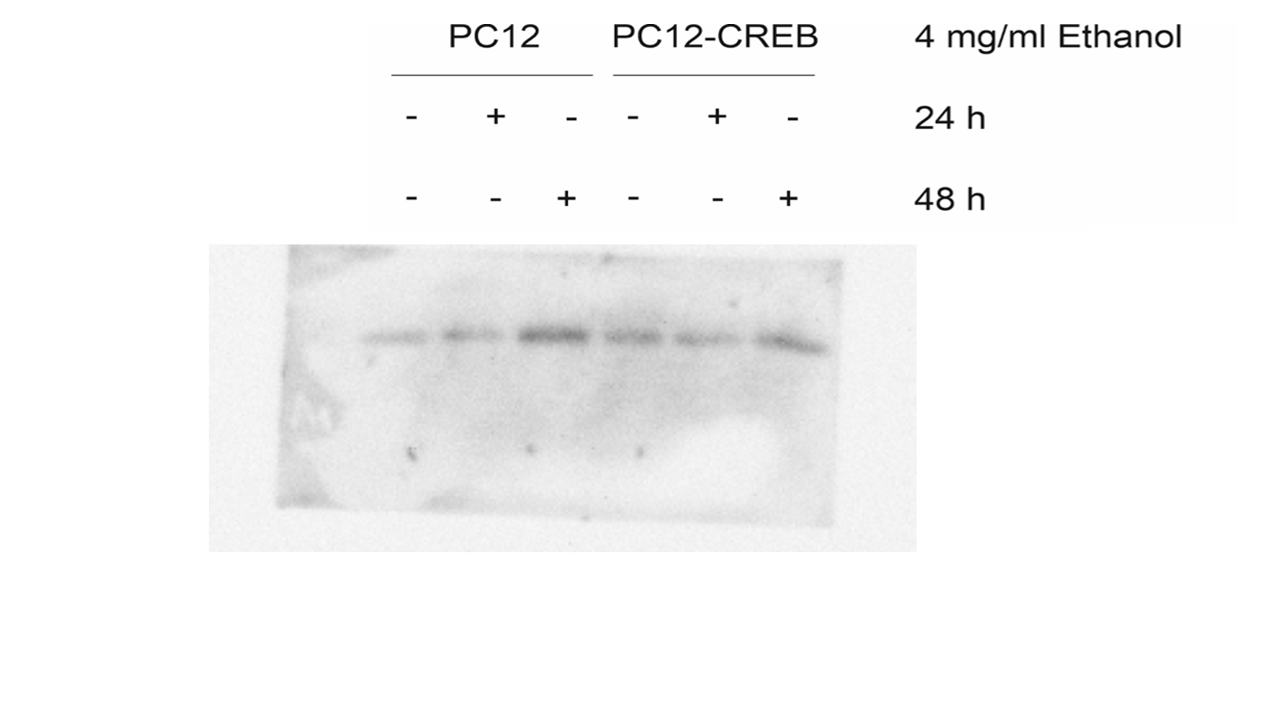

Supplement: Supplementary file 1 [file biology-14-01277-s001.zip › biology-3795769-Supplementary Figure S1/Figure_4b_cleaved_caspase-3.tif]

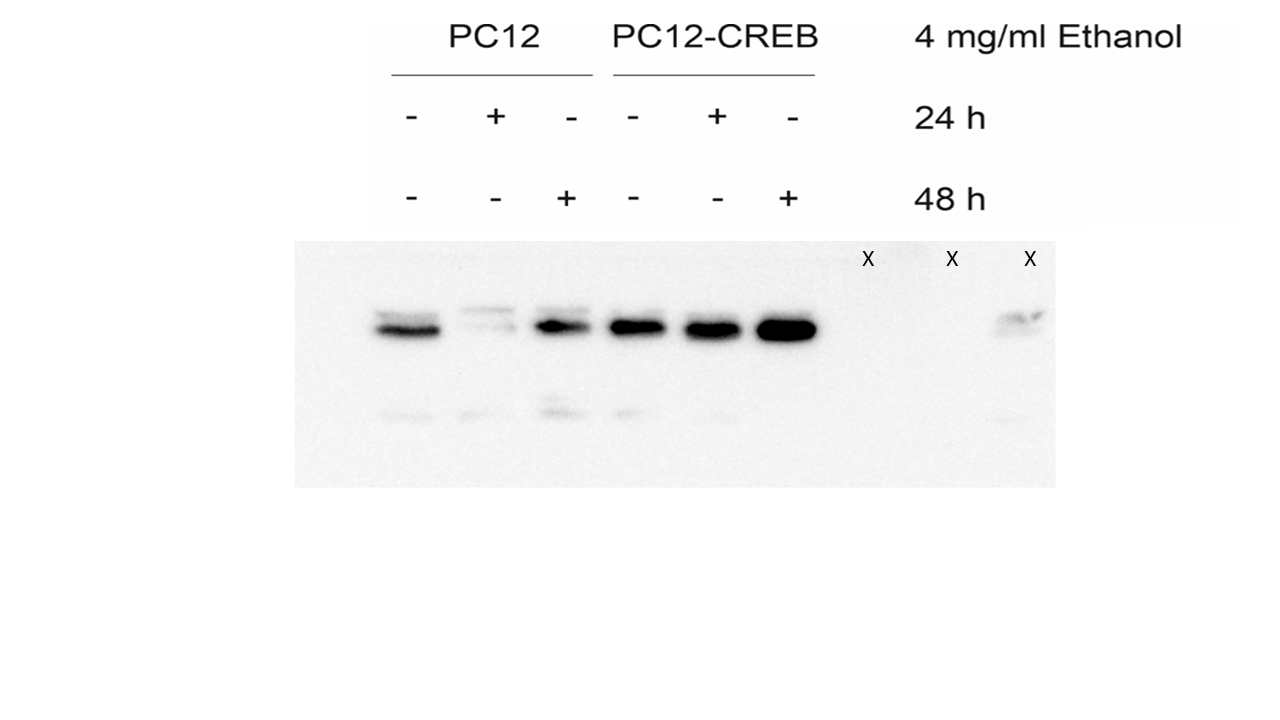

Supplement: Supplementary file 1 [file biology-14-01277-s001.zip › biology-3795769-Supplementary Figure S1/Figure_4b_Mcl1.tif]

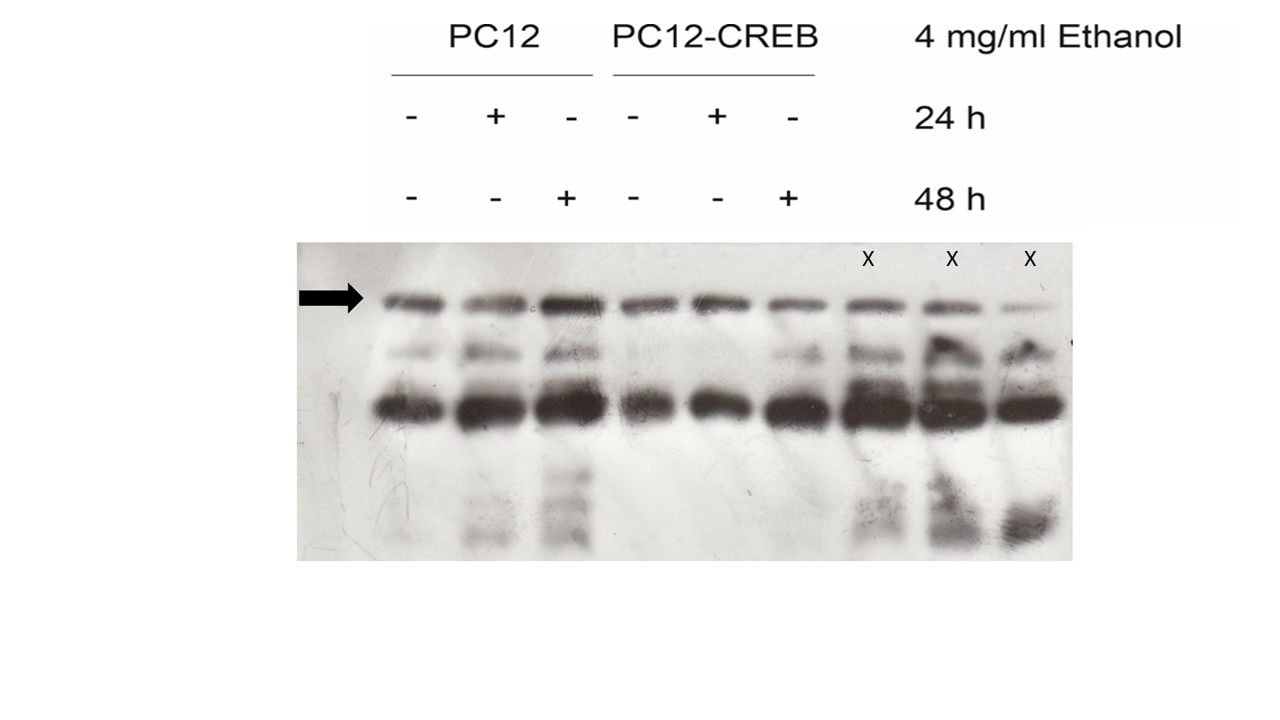

Supplement: Supplementary file 1 [file biology-14-01277-s001.zip › biology-3795769-Supplementary Figure S1/Figure_4b_P-Bad.tif]

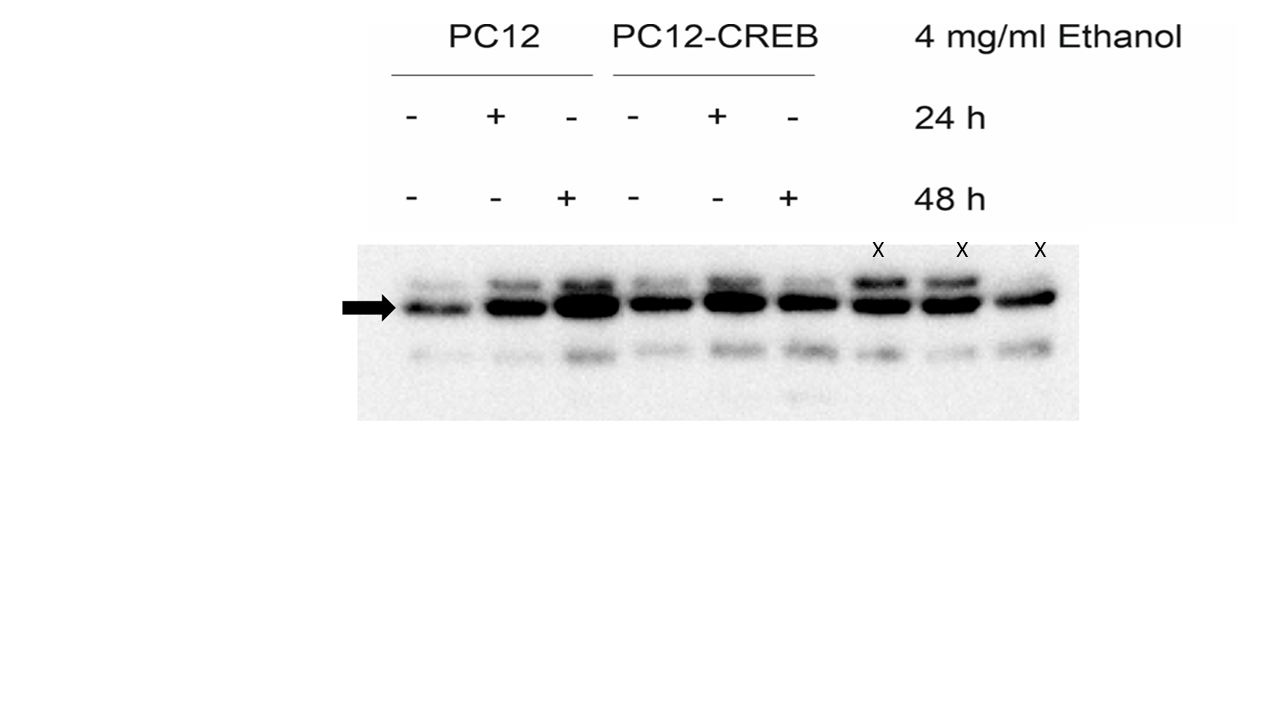

Supplement: Supplementary file 1 [file biology-14-01277-s001.zip › biology-3795769-Supplementary Figure S1/Figure_4b_Puma.tif]
